# Longitudinal Kinetics of Cerebrospinal Fluid and Serum S100B in Patients with Delayed Cerebral Ischemia After Aneurysmal Subarachnoid Hemorrhage

**DOI:** 10.3390/ijms27167274

**Published:** 2026-08-14

**Authors:** Małgorzata Burzyńska, Adrianna Lebiedzińska, Jarosław Kędziora, Katarzyna Smarzewska, Anna Lemańska-Perek, Maciej Miś, Agnieszka Uryga, Barbara Adamik, Waldemar Goździk

**Affiliations:** 1 Clinical Department of Anesthesiology and Intensive Therapy, Faculty of Medicine, Wroclaw Medical University, 50-367 Wroclaw, Poland; malgorzata.burzynska@umw.edu.pl (M.B.);; 2 Clinical Department of Anesthesiology and Intensive Therapy, University Clinical Hospital in Wroclaw, 50-556 Wroclaw, Poland; 3 Clinical Department of Neurosurgery, University Centre for Neurology and Neurosurgery, University Clinical Hospital in Wroclaw, 50-556 Wroclaw, Poland; 4 Department of Biochemistry and Immunochemistry, Wroclaw Medical University, 50-369 Wroclaw, Poland; 5 Department of Biomedical Engineering, Faculty of Fundamental Problems of Technology, Wroclaw University of Science and Technology, 50-377 Wroclaw, Poland

**Keywords:** aneurysmal subarachnoid hemorrhage, neuroinflammation, delayed cerebral ischemia, protein S100B, neuron-specific enolase

## Abstract

S100 calcium-binding protein B (S100B) has been proposed as a biomarker of brain injury after aneurysmal subarachnoid hemorrhage (aSAH), although the clinical utility of serial measurements remains uncertain. This prospective observational study investigated whether longitudinal serum and cerebrospinal fluid (CSF) S100B concentrations are associated with delayed cerebral ischemia (DCI), inflammatory activation, and neurological outcome. Twenty-six consecutive patients with aSAH underwent serial serum and CSF S100B measurements throughout hospitalization. Associations between S100B concentrations, DCI, disease severity, inflammatory biomarkers, and 3-month neurological outcome were analyzed. Admission serum S100B concentrations were significantly higher in patients who subsequently developed DCI (152.6 vs. 21.0 ng/L, *p* = 0.012) and correlated with disease severity and inflammatory biomarkers. Admission serum S100B was associated with DCI and showed good discriminative ability for DCI (AUC 0.79) and unfavorable 3-month outcome (AUC 0.86). Longitudinal analyses showed persistently elevated serum S100B concentrations in patients who developed DCI, while serum and CSF S100B exhibited similar temporal profiles throughout the observation period Serial serum S100B measurements appear to reflect the temporal evolution of secondary brain injury after aSAH and may provide complementary biological information alongside established clinical assessment.

## 1. Introduction

Aneurysmal subarachnoid hemorrhage (aSAH) accounts for approximately 5% of all strokes and remains associated with substantial mortality and long-term disability despite advances in neurocritical care [1,2,3]. Its clinical course is driven by a complex cascade of pathophysiological events involving early brain injury (EBI) and delayed cerebral ischemia (DCI) [4,5,6]. Although temporally distinct, these conditions, EBI and DCI, share several underlying mechanisms including cerebral ischemia, blood–brain barrier (BBB) disruption, neuroinflammation, and microvascular dysfunction. Unlike EBI, which is largely established at the time of hemorrhage, DCI represents a potentially preventable and treatable secondary complication, providing a critical window for therapeutic intervention [4,5,6,7,8,9].

Beyond neurological injury, patients with aSAH frequently develop systemic complications, including pulmonary dysfunction, cardiovascular injury, and infections, driven by sympathetic activation, endothelial dysfunction, and systemic inflammation [10,11,12,13,14,15]. As these processes evolve dynamically, there is growing interest in biomarkers that reflect ongoing secondary brain injury and improve biological characterization of disease evolution.

S100 calcium-binding protein B (S100B) is one of the most extensively investigated biomarkers of acute brain injury [16,17]. It is predominantly released by astrocytes following cellular activation or injury, although it is also expressed by several other neural cell types [17,18,19,20,21,22]. Following BBB disruption, S100B is released into both cerebrospinal fluid (CSF) and peripheral blood, making it an attractive circulating biomarker of central nervous system injury [23,24]. Besides reflecting astroglial damage, extracellular S100B may amplify neuroinflammation through activation of the receptor for advanced glycation end products (RAGE) and damage-associated molecular pattern (DAMP) signaling pathways [17,18,19,25,26].

Accordingly, S100B has been extensively investigated across a broad spectrum of neurological disorders [16,17]. In mild traumatic brain injury, its high sensitivity has led to its incorporation into the Scandinavian guidelines for patient management [27]. In contrast, studies in aSAH have primarily evaluated S100B as a prognostic biomarker, demonstrating associations with hemorrhage severity and unfavorable outcome, whereas its relationship with DCI remains inconsistent [16,28,29,30].

The inconsistency of previous findings may partly reflect substantial heterogeneity in study design, including differences in sampling schedules, biological compartments, patient populations, and definitions of clinical endpoints [16,28,29,30]. While some studies evaluated a single admission or early post-ictal serum measurement, others focused on mean or peak S100B concentrations obtained during hospitalization [28,29,30]. Moreover, angiographic vasospasm, DND, cerebral infarction, and clinically defined DCI have frequently been used interchangeably despite representing distinct, although related, pathophysiological entities [4,31]. Consequently, the temporal relationship between S100B kinetics and clinically defined DCI remains insufficiently characterized [28,29,30].

Furthermore, most previous studies have relied on single early measurements, providing limited insight into the dynamic evolution of astroglial injury during the period of greatest DCI risk [16,28,29,30]. Serial assessment of S100B has the potential to better characterize the temporal evolution of secondary brain injury and clarify whether circulating serum concentrations reflect changes occurring within the central nervous system [17,25]. Therefore, the present study aimed to characterize longitudinal S100B kinetics in paired serum and cerebrospinal fluid samples, evaluate their temporal relationship with DCI, and examine their associations with inflammatory activation, disease severity, and neurological outcome. Rather than establishing S100B as an independent prognostic marker, this exploratory study sought to provide a better biological understanding of longitudinal S100B dynamics after aSAH.

## 2. Results

### 2.1. Study Population and Baseline Characteristics

**Figure 1:**
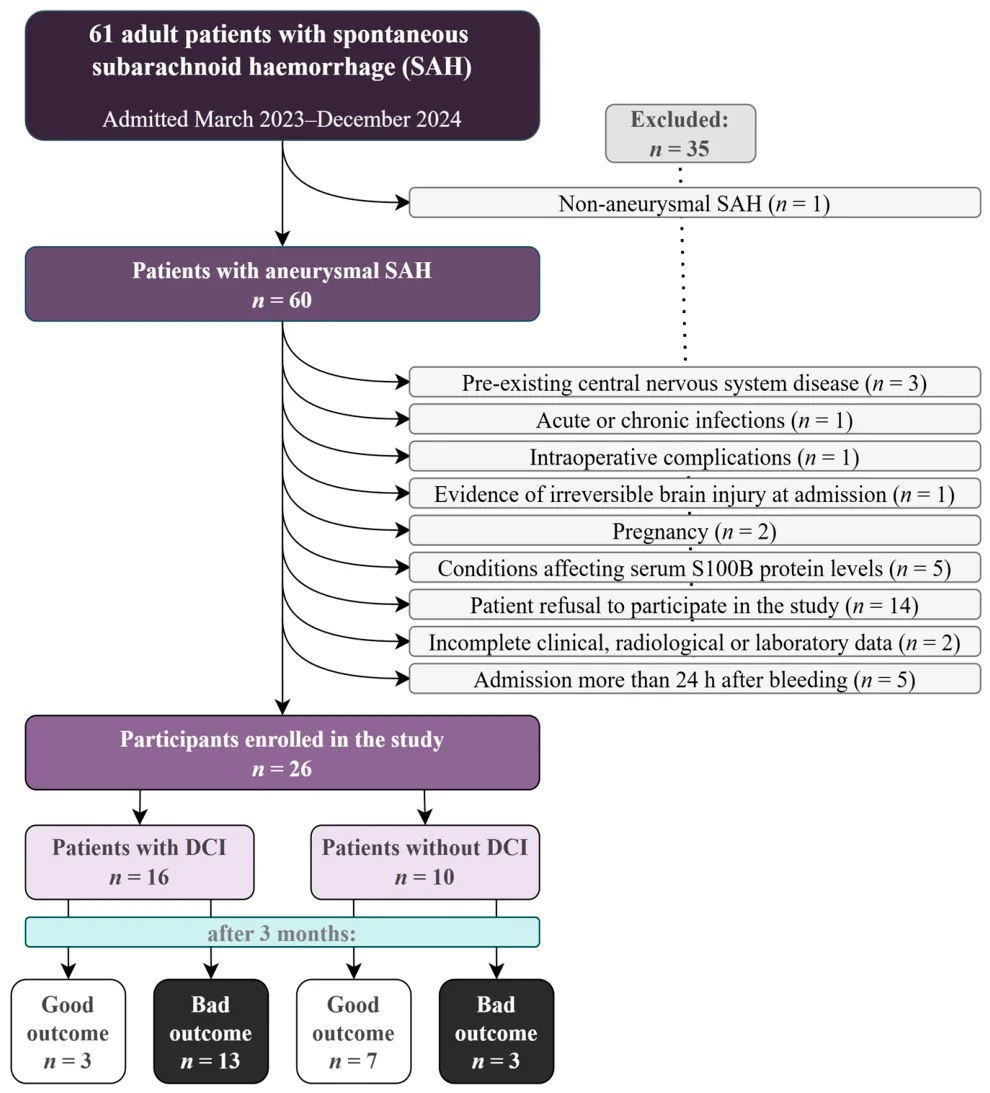
Flowchart of patient selection and study cohort formation.

The study included 26 patients with aSAH. DCI developed in 16 patients (61.5%), and unfavorable 3-month neurological outcome was observed in 16 patients (61.5%). Patient selection and study cohort formation are summarized in Figure 1. Baseline demographic, clinical, laboratory, and treatment-related characteristics are summarized in Table 1; only variables showing significant between-group differences are highlighted below.

Patients who developed DCI had significantly higher admission serum S100B concentrations measured within the first 24 h after admission (152.57 [38.46–540.61] vs. 21.04 [16.02–76.45] ng/L, *p* = 0.012), while admission interleukin-6 (IL-6) concentrations showed a similar trend (23.35 [16.00–36.90] vs. 10.95 [6.92–20.50] pg/mL, *p* = 0.055). The DCI group also presented with greater disease severity, reflected by higher APACHE II scores (22.5 [17.0–30.5] vs. 11.5 [9.0–20.0], *p* = 0.045) and more frequent impairment of motor responsiveness at admission defined as motor Glasgow Coma Scale (motor GCS) < 6: 75.0% vs. 30.0%, *p* = 0.043.

Patients with DCI more frequently underwent endovascular aneurysm treatment, whereas microsurgical clipping was more common among patients without DCI. Infectious complications and focal neurological deficits also occurred more frequently during hospitalization in the DCI group. No significant between-group differences were observed for demographic characteristics, vascular risk factors, admission hemodynamics, aneurysm location, baseline radiological severity scales, renal function, or length of hospital stay. Clinical outcomes, vasospasm characteristics, autoregulatory indices, and in-hospital complications according to DCI status are summarized in Table 2.

At hospital discharge, 42.3% of patients were discharged home, 26.9% required inpatient rehabilitation, 7.7% were transferred to long-term care facilities, and 23.1% died during hospitalization. All in-hospital deaths occurred in patients with DCI. The median time to death was 13.5 days (IQR 11.5–14.75 days; range 9–20 days), and Kaplan–Meier analysis demonstrated significantly reduced survival in the DCI group (log-rank *p* = 0.039). Functional outcomes were significantly worse among patients with DCI, with unfavorable modified Rankin Scale (mRS) scores observed more frequently both at discharge (94% vs. 40%, *p* = 0.005) and at 3 months (81% vs. 30%, *p* = 0.015).

### 2.2. Association Between Admission Serum S100B and Disease Severity

**Figure 2:**
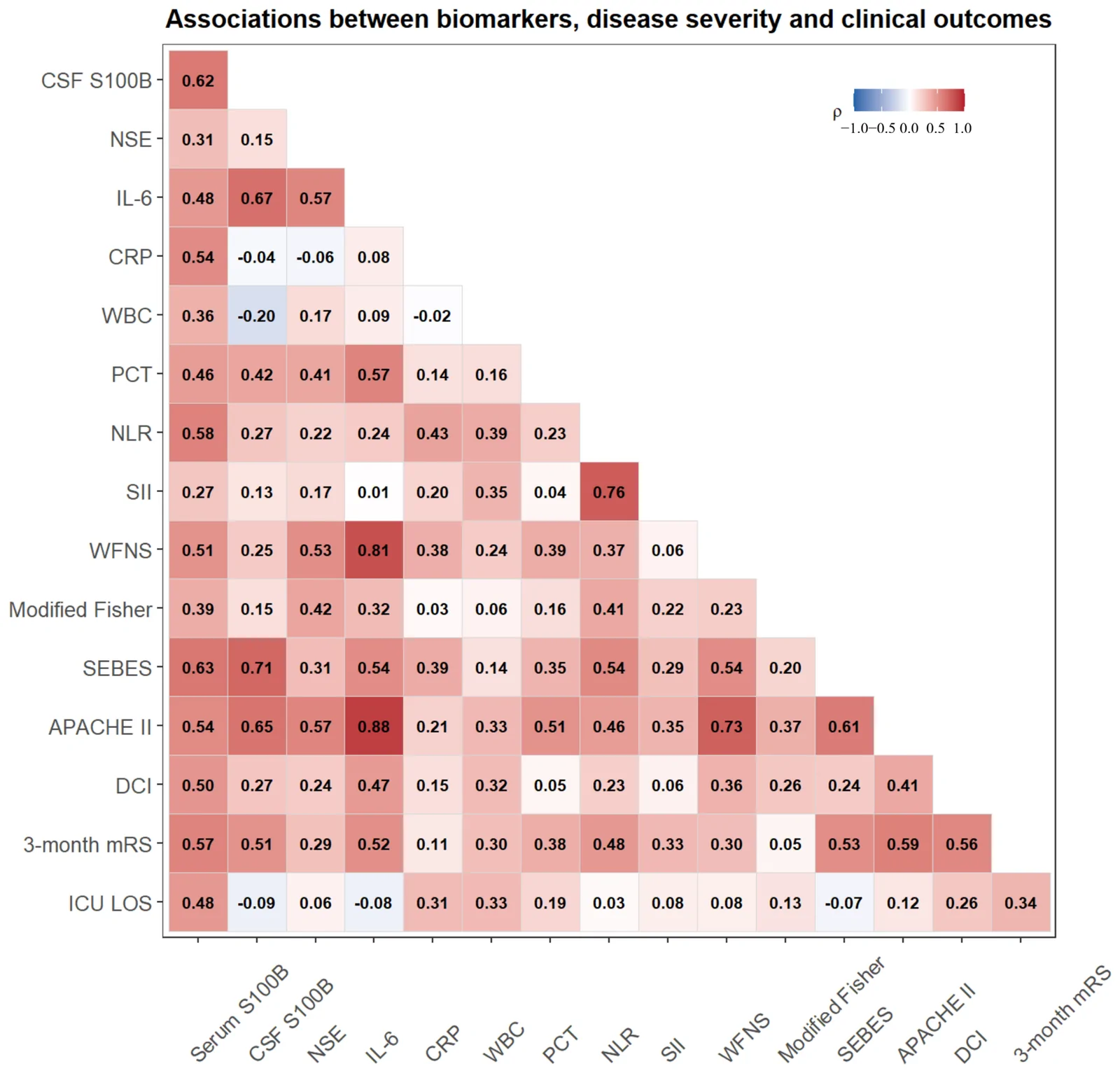
Associations between biomarkers, disease severity, and clinical outcomes.

Admission serum S100B concentrations correlated significantly with multiple measures of disease severity, including APACHE II score, Hunt–Hess grade, WFNS classification, the Subarachnoid Hemorrhage Early Brain Edema Score (SEBES) score, and VASOGRADE category (Figure 2). Higher S100B concentrations were also associated with lower GCS and motor GCS scores.

In Cox proportional hazards analysis, higher admission S100B concentrations showed a trend toward an increased risk of in-hospital mortality, although the association did not reach statistical significance (HR 1.001, *p* = 0.087). Admission S100B concentrations were not associated with serum creatinine levels, suggesting that renal function did not substantially influence circulating S100B concentrations in this cohort. In multivariable logistic regression analysis, admission serum S100B was not independently associated with unfavorable 3-month neurological outcome after adjustment for age, WFNS grade, and Fisher score (*p* = 0.81). Among the clinical variables included in the model, only age remained independently associated with poor functional outcome (*p* = 0.024). The baseline clinical prediction model incorporating age, WFNS grade, and Fisher score demonstrated an AUC of 0.83, and addition of serum S100B did not significantly improve outcome prediction (DeLong test, *p* = 0.48).

Heatmap showing pairwise Spearman correlation coefficients (ρ) between admission biomarkers, disease severity scales, and clinical outcomes. Positive correlations are displayed in red and negative correlations in blue, with color intensity proportional to correlation strength. Numerical values within cells represent Spearman correlation coefficients. S100B, S100 calcium-binding protein B; CSF, cerebrospinal fluid; NSE, neuron-specific enolase; IL-6, interleukin-6; CRP, C-reactive protein; WBC, white blood cell count; WFNS, World Federation of Neurosurgical Societies grade; SEBES, Subarachnoid Hemorrhage Early Brain Edema Score; APACHE II, Acute Physiology and Chronic Health Evaluation II; DCI, delayed cerebral ischemia; mRS, modified Rankin Scale; ICU LOS, intensive care unit length of stay.

### 2.3. Association of Admission and Longitudinal Serum S100B Concentrations with DCI and Neurological Outcome

**Figure 3:**
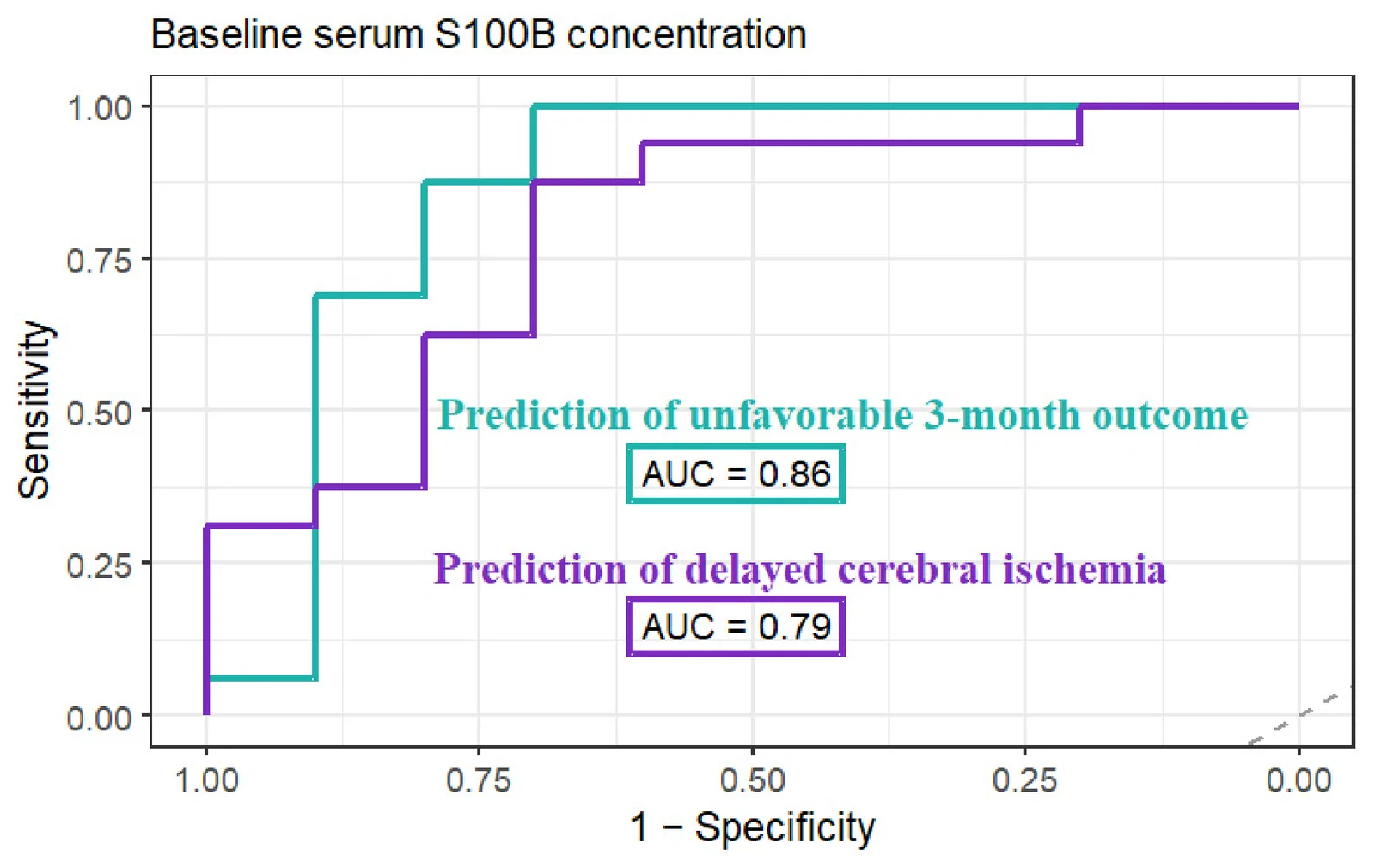
Receiver operating characteristic (ROC) curves for admission serum S100B concentration.

Admission serum S100B concentrations measured within the first 24 h after ictus were associated with both DCI and unfavorable 3-month neurological outcome and showed good discriminative ability in ROC analyses. The diagnostic performance of admission serum S100B is illustrated by the ROC curves shown in Figure 3. ROC analysis showed an AUC of 0.79 for DCI prediction, with an optimal cutoff value of 28.54 ng/L corresponding to 87.5% sensitivity and 70% specificity. For prediction of unfavorable 3-month outcome, the AUC was 0.86 (95% CI 0.67–1.00), with an optimal cutoff value of 22.25 ng/L, providing 100% sensitivity and 70% specificity.

**Figure 4:**
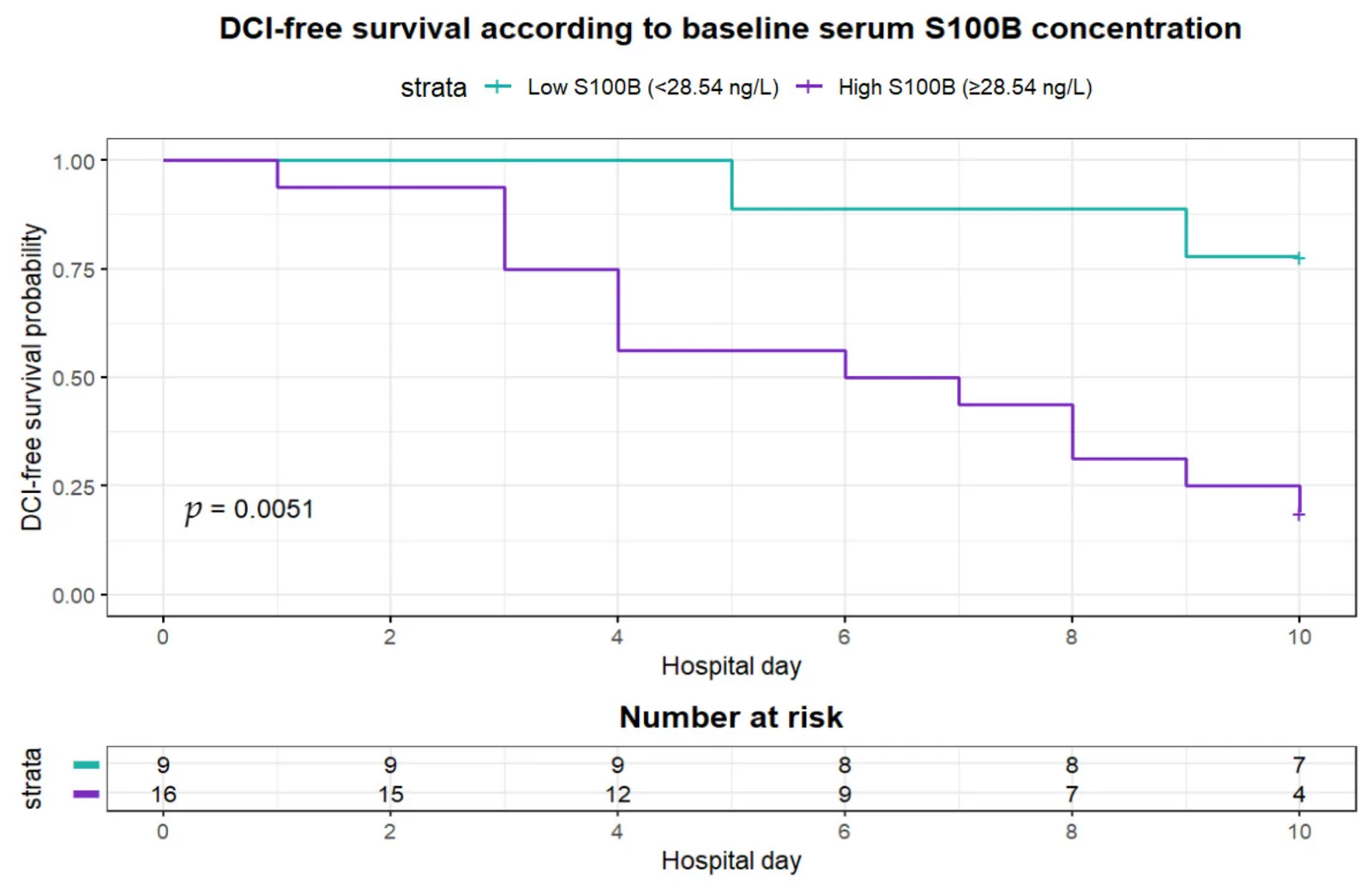
Delayed cerebral ischemia (DCI)-free survival according to admission serum S100B concentration.

Time-to-event analyses further demonstrated that higher admission serum S100B concentrations were associated with earlier DCI occurrence during hospitalization. In Cox proportional hazards analysis, each 100 ng/L increase in admission S100B concentration was associated with a 15.7% increase in DCI hazard (HR 1.16, 95% CI 1.02–1.31, *p* = 0.024). Similarly Kaplan–Meier analysis using the ROC-derived cutoff of 28.54 ng/L demonstrated significantly reduced DCI-free survival among patients with elevated admission serum S100B concentrations (log-rank *p* = 0.005). Kaplan–Meier curves illustrating DCI-free survival according to the ROC-derived admission serum S100B cutoff are presented in Figure 4.

Peak serum S100B concentrations during hospitalization were also associated with DCI occurrence (logistic regression, *p* = 0.049). ROC analysis of peak S100B demonstrated an AUC of 0.87 for DCI prediction. Analysis of the last available serum S100B measurement before DCI onset, ROC analysis yielded an AUC of 0.81, with an optimal cutoff value of 33.2 ng/L corresponding to 55.6% sensitivity and 100% specificity. Because peak concentrations frequently occurred after DCI onset, these analyses should be interpreted as describing the evolution of biomarker levels rather than their predictive performance.

**Figure 5:**
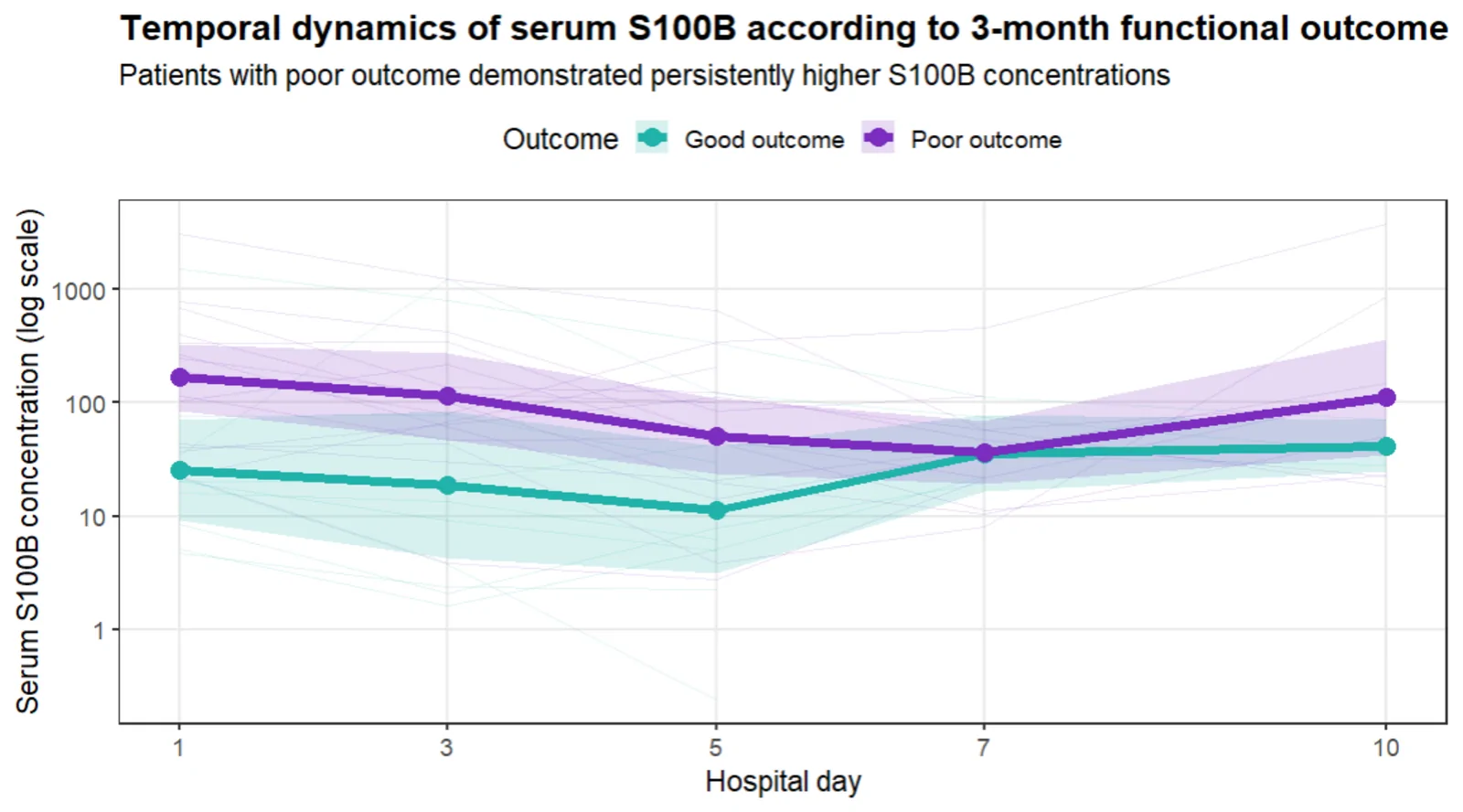
Temporal dynamics of serum S100B according to 3-month functional outcome.

Longitudinal mixed-effects modeling demonstrated a significant overall decline in serum S100B concentrations throughout hospitalization (β = −0.44, *p* < 0.001). Nevertheless, patients who developed DCI maintained consistently higher serum S100B concentrations over time than those without DCI (β = 2.59, *p* = 0.003), whereas temporal trajectories did not differ significantly between groups (interaction *p* = 0.397). To assess the robustness of this association, additional mixed-effects models were fitted adjusting for potential confounders. The association between DCI and higher longitudinal serum S100B concentrations remained significant after adjustment for infectious complications (*p* = 0.008). Infectious complications themselves were not independently associated with longitudinal serum S100B concentrations (*p* = 0.113). In an additional sensitivity analysis adjusting for treatment modality, DCI remained significantly associated with longitudinal serum S100B concentrations (β = 2.50, *p* < 0.001), whereas treatment modality itself was not significantly associated with serum S100B concentrations (β = −1.38, *p* = 0.061). These longitudinal patterns according to 3-month functional outcome are ilustrated in Figure 5.

#### Pre-/Peri-/Post-DCI

Compared with patients who did not develop DCI, serum S100B concentrations were already significantly higher during the Pre-DCI phase (β = 1.62, *p* = 0.002), remained elevated during the Peri-DCI phase (β = 1.75, *p* = 0.012), and were highest during the Post-DCI phase (β = 2.54, *p* < 0.001), after adjustment for hospital day. However, among patients who developed DCI, no significant increase in serum S100B concentrations was observed between the Pre-DCI and Peri-DCI phases (*p* = 0.798), although serum S100B concentrations tended to be higher during the Post-DCI phase (*p* = 0.084). A separate mixed-effects model including an interaction between DCI status and hospital day demonstrated persistently higher serum S100B concentrations in patients who developed DCI (*p* = 0.012), whereas differences in temporal trajectories showed only a trend toward significance (interaction *p* = 0.073).

Additional analyses addressing the potential influence of renal function further supported the robustness of these findings. Adjustment for admission serum creatinine did not materially alter the association between DCI and longitudinal serum S100B concentrations. Likewise, serum creatinine measured on day 5 was not independently associated with concurrent serum S100B concentrations (*p* = 0.434) and did not differ between patients with and without DCI (*p* = 0.356). Although serum creatinine decreased significantly between admission and day 5 (*p* = 0.003), adjustment for day-5 creatinine did not materially alter the association between DCI and longitudinal serum S100B concentrations (β = 1.96, *p* = 0.002). These findings suggest that impaired renal clearance is unlikely to fully account for the observed longitudinal serum S100B profile.

A similar pattern was observed among patients with unfavorable 3-month neurological outcome, who exhibited persistently higher serum S100B concentrations without differences in temporal trajectories (interaction *p* = 0.869). TCD-defined vasospasm showed a trend toward differential longitudinal S100B trajectories (interaction *p* = 0.066). Exploratory analyses of cumulative longitudinal serum S100B burden demonstrated greater overall biomarker exposure among patients who developed DCI and those with unfavorable neurological outcomes. However, these associations were attenuated after adjustment for clinical severity measures.

ROC analysis evaluating the performance of admission serum S100B for prediction of delayed cerebral ischemia (DCI) and unfavorable 3-month functional outcome (modified Rankin Scale 3–6). The area under the curve (AUC) was 0.79 for DCI and 0.86 for unfavorable 3-month outcome.

Kaplan–Meier curves comparing DCI-free survival in patients with admission serum S100B concentrations below and above the ROC-derived cutoff value of 28.5 ng/L. The *p*-value was calculated using the log-rank test.

Longitudinal trajectories of serum S100B concentrations measured on hospital days 1, 3, 5, 7, and 10 according to 3-month functional outcome. Individual patient trajectories are shown as thin lines, whereas thick lines and shaded areas represent group median values and interquartile ranges. Serum S100B concentrations are presented on a logarithmic scale.

### 2.4. Comparison of Serum and Cerebrospinal Fluid S100B Kinetics

Longitudinal comparison of serum and CSF S100B concentrations demonstrated substantially higher S100B levels in CSF than in serum throughout hospitalization (β = 4.26, *p* < 0.001). Concentrations declined over time in both compartments (β = −0.21, *p* < 0.001), while temporal trajectories remained parallel over time (with no significant compartment-by-time interaction *p* = 0.904).

Spearman correlation analyses performed separately for each sampling day demonstrated a significant positive correlation between paired serum and CSF S100B concentrations on day 1 (ρ = 0.621, *p* = 0.024). Positive but non-significant correlations were observed on days 3 (ρ = 0.373), 5 (ρ = 0.421), 7 (ρ = 0.285), and 10 (ρ = 0.400), which was likely attributable to the limited number of paired samples available at later time points.

In a linear mixed-effects model accounting for repeated measurements and sampling day, CSF sampling route was not significantly associated with CSF S100B concentrations (all *p* > 0.30), whereas sampling day remained the only variable independently associated with S100B concentrations (*p* = 0.023).

Similarly, infectious complications and treatment modality were not independently associated with longitudinal CSF S100B concentrations, whereas sampling day remained the only significant predictor.

### 2.5. Association Between Serum S100B, Inflammation, and Systemic Complications

**Figure 6:**
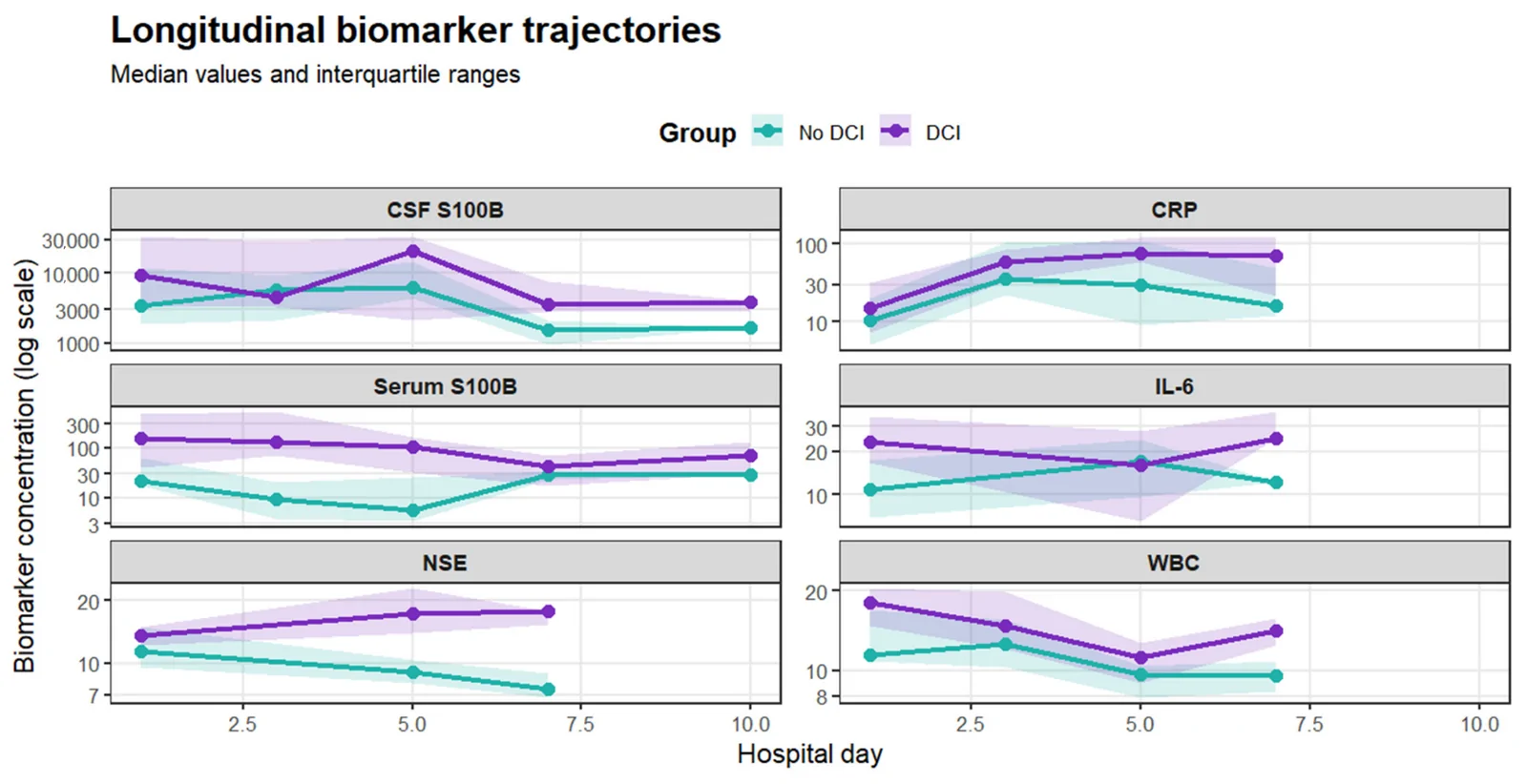
Longitudinal biomarker trajectories according to delayed cerebral ischemia status. Temporal profiles of cerebrospinal fluid (CSF) S100B, serum S100B, neuron-specific enolase (NSE), interleukin-6 (IL-6), C-reactive protein (CRP), and white blood cell count (WBC) according to delayed cerebral ischemia (DCI) status. Solid lines represent median values and shaded areas indicate interquartile ranges. Biomarker concentrations are presented on a logarithmic scale.

Admission serum S100B concentrations correlated positively with several inflammatory biomarkers, including neutrophil-to-lymphocyte ratio (NLR) (ρ = 0.58, *p* = 0.004; FDR-adjusted *p* = 0.013), C-reactive protein (CRP) (ρ = 0.54, *p* = 0.004; FDR-adjusted *p* = 0.013), and procalcitonin (PCT) (ρ = 0.46, *p* = 0.017; FDR-adjusted *p* = 0.034). A nominal positive correlation was also observed with IL-6 (ρ = 0.48, *p* = 0.044), although this association did not remain statistically significant after Benjamini–Hochberg correction for multiple comparisons (FDR-adjusted *p* = 0.066). No significant correlations were observed for white blood cell (WBC) count or systemic immune-inflammation index (SII) (Figure 6).

Longitudinal analyses demonstrated significantly different CRP trajectories among patients who developed DCI (interaction β = 0.28, *p* = 0.018), indicating persistently higher CRP concentrations over time. In contrast, WBCs declined during hospitalization without significant differences according to DCI status. In multivariable exploratory analyses, WBC emerged as the strongest independent inflammatory correlate identified in the exploratory model of serum S100B (*p* = 0.007).

Although infectious complications occurred after completion of the predefined biomarker sampling period, patients who subsequently developed infections exhibited higher admission and longitudinal serum S100B concentrations than those without infection (both *p* = 0.044). In contrast, serum S100B concentrations were not significantly associated with neurogenic pulmonary edema or cardiac complications.

In CSF, sampling day remained significantly associated with S100B concentrations (*p* = 0.014), while neither DCI nor infectious complications reached statistical significance. These findings suggest that temporal changes in CSF S100B were primarily driven by time from ictus rather than by DCI or systemic complications.

### 2.6. Prognostic Performance of Neuron-Specific Enolase

Admission neuron-specific enolase (NSE) concentrations measured on day 1 did not differ between patients with and without DCI (*p* = 0.263). In contrast day-5 NSE concentrations were significantly higher in patients who developed DCI (*p* = 0.0007). Changes in NSE concentrations between days 1 and 5 showed a trend toward significance (*p* = 0.068).

ROC analysis demonstrated lower discriminative performance for NSE than for admission serum S100B (AUC 0.64 vs. 0.79), although the difference between biomarkers was not statistically significant (DeLong test, *p* = 0.27).

### 2.7. Cerebral Autoregulation

Dynamic cerebral autoregulation assessed using the autoregulation index (ARI) and the modified mean flow index (Mxa) was not significantly associated with DCI, vasospasm, or 3-month neurological outcome. Likewise, no significant correlations were observed between serum S100B concentrations and either autoregulatory index.

## 3. Discussion

### 3.1. Clinical Associations of Serum S100B After aSAH

The present study evaluated longitudinal serum and CSF S100B kinetics following aSAH and identified significant associations between serum S100B concentrations, DCI, inflammatory activation, vasospasm burden, and neurological outcome. Admission serum S100B concentrations measured within the first 24 h after ictus correlated with multiple established markers of disease severity, whereas patients who subsequently developed DCI exhibited persistently elevated serum S100B concentrations throughout hospitalization. These findings suggest that serum S100B reflects both the severity of the initial hemorrhagic injury and the subsequent evolution of secondary brain injury. Its close association with established clinical severity measures may explain why the association with long-term functional outcome was attenuated after adjustment for conventional prognostic variables.

Kaplan–Meier analyses demonstrated significantly shorter DCI-free survival among patients with elevated admission S100B concentrations, indicating that early astroglial injury may be associated with subsequent secondary ischemic complications. Similarly, patients who developed DCI exhibited significantly reduced overall survival, with all in-hospital deaths occurring in the DCI group, highlighting the major contribution of secondary ischemic injury on prognosis after aSAH.

The present findings extend our previous observations demonstrating the prognostic value of S100B in patients with aSAH [31]. Although Cox proportional hazards analyses suggested a possible association between elevated serum S100B concentrations and mortality, statistical significance was not consistently achieved, likely because of the limited number of deaths and the relatively small cohort size. Nevertheless, the clinical relevance of S100B after aSAH has been consistently demonstrated in previous studies. Azurmendi et al. showed that serial S100B measurements were associated with long-term neurological outcome [32]. Kellermann et al. identified serum S100B concentrations exceeding 0.7 μg/L as a marker of extremely poor prognosis [33], whereas Zhou et al. and Sánchez-Peña et al. reported associations between elevated S100B concentrations and unfavorable neurological outcome, including severe disability and mortality [28,34,35]. Quintard et al. further demonstrated that peripheral S100B concentrations measured during the first week after aSAH were associated with poor neurological outcome, with the highest predictive performance observed on day 5 [36].

In contrast, Kiiski et al. found no association between plasma S100B concentrations and 6-month neurological outcome despite evaluating several circulating biomarkers, including S100B and NSE [37]. They additionally reported higher S100B concentrations in patients with less severe initial presentation and suggested that S100B elevation may partly represent an adaptive response to brain injury. These discrepancies may reflect differences in study design, patient populations, sampling protocols, and outcome definitions. Furthermore, the concentration-dependent biological effects of S100B, with neurotrophic properties at physiological concentrations and potentially detrimental effects at higher levels, emphasize the complexity of its role in secondary brain injury [17,19].

Overall, these findings suggest that the longitudinal S100B assessment may provide complementary biological information regarding the dynamic evolution of secondary brain injury after aSAH [38].

### 3.2. Longitudinal S100B Dynamics and Secondary Brain Injury

One of the main findings of the present study was the persistent elevation of serum S100B concentrations in patients who developed DCI. Additional mixed-effects analyses performed relative to the timing of DCI demonstrated that serum S100B concentrations were already significantly elevated before the clinical onset of DCI and remained persistently higher thereafter. Importantly, serial assessment may distinguish a transient increase related predominantly to the initial hemorrhagic insult from persistently elevated concentrations that may reflect ongoing secondary brain injury during the period of greatest DCI risk [39,40]. In contrast, no abrupt increase in serum S100B concentrations was observed at the time of DCI onset, suggesting that S100B reflects the overall burden of secondary brain injury rather than serving as an acute marker of DCI occurrence. Despite these differences, temporal trajectories remained broadly similar, suggesting that DCI is characterized by sustained S100B elevation rather than fundamentally altered biomarker kinetics. The association between longitudinal serum S100B and DCI remained significant after adjustment for infectious complications and treatment modality, indicating that the observed relationship was not explained by either factor. Treatment modality itself was not significantly associated with longitudinal serum S100B concentrations (β = −1.38, *p* = 0.061). Because infectious complications typically develop later during hospitalization, they are more likely to represent downstream clinical events rather than baseline determinants of early S100B release. The persistence of the association after adjustment therefore supports the interpretation that elevated longitudinal S100B concentrations primarily reflect secondary brain injury rather than infection itself.

Additional sensitivity analyses further strengthened these observations. The association between longitudinal serum S100B concentrations and DCI remained statistically significant after adjustment for baseline disease severity using either the APACHE II score or WFNS grade. While higher APACHE II scores were independently associated with elevated longitudinal serum S100B concentrations, adjustment for either severity scale did not materially alter the association between DCI and S100B. These findings suggest that the persistent elevation of serum S100B in patients who develop DCI cannot be explained solely by differences in baseline clinical severity. Given the relatively short biological half-life of circulating S100B [17,19], persistently elevated concentrations are less likely to reflect residual release from the initial hemorrhage alone and may instead indicate ongoing astroglial injury associated with secondary pathophysiological processes.

The biological rationale for serial S100B assessment reflects the dynamic evolution of brain injury after aSAH. Whereas admission S100B concentrations primarily reflect the severity of the initial hemorrhagic insult and early astroglial injury, secondary complications such as DCI evolve over several days. Consequently, a single early measurement may not adequately capture these later pathophysiological processes, whereas longitudinal assessment provides insight into the temporal evolution of secondary brain injury [16]. This evolving secondary injury involves dynamic interactions between astrocytes and microglia, providing a biological framework for interpreting longitudinal changes in astroglial biomarkers such as S100B [41]. Accordingly, serial assessment may provide additional biological insight into evolving secondary brain injury, although our findings do not demonstrate superior prognostic performance compared with admission measurements. Accordingly, this exploratory study should not be interpreted as demonstrating incremental prognostic value of serial S100B measurements beyond established clinical predictors. Rather, the present findings support longitudinal S100B assessment primarily as a complementary biomarker reflecting the temporal evolution of secondary brain injury.

Similarly, patients with vasospasm exhibited persistently elevated serum S100B concentrations and slower biomarker normalization, supporting the concept that prolonged S100B elevation reflects ongoing neurovascular injury. Together with the persistent elevation of serum S100B concentrations over time, these findings further support serial S100B assessment as a complementary component of multimodal risk stratification after aSAH.

These observations are consistent with the growing role of S100B across acute neurological disorders. In mild traumatic brain injury, S100B has been incorporated into the Scandinavian guidelines because of its high sensitivity despite limited specificity [27]. Similarly, following cardiac arrest, current international recommendations advocate the use of S100B only as part of a multimodal prognostic strategy rather than as an isolated predictor [42,43,44]. Collectively, these findings suggest that serial S100B monitoring may serve as an adjunct to established clinical, imaging, and neurophysiological assessment rather than a replacement for current prognostic strategies.

In contrast, we did not find significant associations between cerebral autoregulation assessed using ARI and Mxa and DCI, vasospasm, neurological outcome, or serum S100B concentrations. Several factors may explain these findings. ARI and Mxa primarily reflect global autoregulatory capacity and may therefore fail to detect focal cerebrovascular disturbances underlying DCI. Moreover, intermittent measurements may have missed transient episodes of autoregulatory failure preceding ischemic events, while the relatively small sample size further limited statistical power.

Our previous study demonstrated an association between S100B concentrations and cerebral autoregulation assessed using the continuously monitored tissue oxygenation index (TOx) derived from near-infrared spectroscopy [45]. The discrepancy with the present findings may therefore reflect methodological differences between continuous TOx monitoring and intermittent ARI/Mxa assessment. Previous studies investigating the relationship between cerebral autoregulation, vasospasm, and DCI have likewise produced conflicting results [46,47,48,49]. Similarly, Moritz et al., Bellapart et al., Jung et al., and Lai et al. reported limited utility of S100B for identifying CV [50,51,52,53].

Taken together, these findings suggest that intermittent assessment of cerebral autoregulation using ARI and Mxa may have limited utility for identifying patients at risk of DCI. Larger prospective studies integrating continuous autoregulatory monitoring with serial biomarkers of brain injury and blood–brain barrier dysfunction are warranted.

### 3.3. Serum Versus CSF S100B: Is CSF Sampling Necessary?

One clinically relevant question is whether repeated CSF sampling provides additional information beyond serial serum S100B measurements. In the present study, S100B concentrations were consistently higher in CSF than in serum, reflecting the predominantly astroglial origin and intracranial release of the protein after aSAH. Despite these higher absolute concentrations, serum and CSF S100B showed parallel temporal profiles, with no significant compartment-by-time interaction (*p* = 0.904), consistent with the hypothesis that peripheral S100B measurements may reflect intracranial biomarker dynamics. These findings suggest that serial serum S100B measurements may represent a less invasive surrogate for longitudinal assessment of intracranial biomarker dynamics while avoiding the invasiveness and logistical limitations of repeated CSF sampling [28].

Although CSF was collected using different clinically indicated access routes, we found no evidence that the recorded sampling route significantly influenced longitudinal CSF S100B concentrations. Nevertheless, this finding should be interpreted cautiously because the choice of sampling route was not randomized and the number of ventricular samples was limited. Future prospective studies using standardized CSF sampling protocols are warranted to further validate these observations.

### 3.4. Inflammatory Biomarkers and Secondary Brain Injury

Among the inflammatory biomarkers evaluated, CRP showed the strongest and most consistent longitudinal association with DCI. Admission serum S100B concentrations correlated with CRP, NLR, PCT, and nominally with IL-6, whereas WBC emerged as the strongest independent inflammatory correlate in exploratory multivariable analyses. Correlations with NLR, CRP, and PCT remained significant after FDR correction, whereas the association with IL-6 should be interpreted cautiously because it did not remain significant after adjustment for multiple comparisons. Although SII showed only a weak, non-significant correlation with S100B in the present cohort, previous studies suggest that it remains a clinically relevant marker of systemic inflammatory activation after aSAH. These findings are consistent with recent studies linking inflammatory activation with secondary brain injury after aSAH [41,54].

Because infectious complications are common after aSAH and may influence circulating inflammatory biomarkers, we performed additional analyses adjusting for infection. These analyses did not demonstrate an independent association between infectious complications and longitudinal serum or CSF S100B concentrations, suggesting that infection alone is unlikely to explain the observed biomarker kinetics. Importantly, none of the patients had an active clinically diagnosed infection or sepsis during the predefined biomarker sampling period, and all infectious complications were diagnosed only after completion of serial S100B measurements. Higher early S100B concentrations observed in patients who subsequently developed infections therefore likely reflect greater initial neurological injury and a more complicated clinical course rather than infection itself. Previous studies suggest that systemic infection may contribute to secondary brain injury through endothelial dysfunction, microvascular impairment, and amplification of neuroinflammatory responses [55,56,57,58,59]. Conversely, severe EBI may itself predispose patients to subsequent infection through prolonged intensive care treatment, mechanical ventilation, and stroke-induced immunosuppression. Because the temporal sequence of infectious complications relative to DCI could not be established in every patient, the direction of this association remains uncertain and should be interpreted cautiously.

Together, these findings support an association between astroglial injury and systemic inflammatory responses during the evolution of secondary brain injury after aSAH. Following aneurysm rupture, blood breakdown products within the subarachnoid space activate resident microglia and initiate a complex inflammatory cascade involving cytokine release, leukocyte recruitment, endothelial activation, and BBB disruption [15,41]. Within this pathophysiological context, elevated serum S100B concentrations may reflect not only astroglial injury but also ongoing neuroinflammatory activation accompanying delayed ischemic complications. Although these mechanisms are biologically plausible and supported by experimental studies, they were not directly investigated in the present study. Therefore, the observed associations between serum S100B, inflammatory biomarkers, and DCI should not be interpreted as evidence of a causal role of S100B in secondary brain injury. Instead, elevated S100B may represent a bystander marker of astroglial injury and BBB disruption, reflecting the severity of the underlying brain injury rather than directly mediating inflammatory processes. Further experimental and translational studies are required to determine whether S100B actively contributes to secondary brain injury or primarily reflects its severity.

NLR and SII represent practical biomarkers of systemic immune activation. NLR reflects the balance between activation of innate immunity and suppression of lymphocyte-mediated adaptive responses, whereas SII integrates platelet, neutrophil, and lymphocyte counts to capture both inflammatory and thrombo-inflammatory activity. Elevated admission NLR has consistently been associated with DCI, unfavorable neurological outcome, and mortality after aSAH [59]. Similarly, increased SII has been linked to greater hemorrhage severity, higher risk of DCI, and poor functional outcome, supporting the contribution of systemic immune activation and platelet-mediated vascular dysfunction to secondary brain injury [60,61,62,63]. Consistent with these observations, Shi et al. identified elevated admission NLR as an independent predictor of DCI [64], whereas Tian et al. and Cai et al. reported associations between higher NLR values, greater neurological severity, and increased risk of complications [65,66]. These findings have subsequently been confirmed by several meta-analyses [60,65]. Likewise, elevated IL-6, CRP, and WBC concentrations have consistently been associated with DCI development and poor neurological outcome after aSAH [67,68,69,70]. The present finding that CRP exhibited the strongest longitudinal association with DCI further supports the contribution of sustained systemic inflammatory activation to delayed secondary brain injury.

Additional analyses addressing the potential influence of renal function further strengthened these findings. Adjustment for admission serum creatinine did not materially alter the association between DCI and longitudinal serum S100B concentrations, and serum creatinine measured on day 5 was neither independently associated with concurrent serum S100B concentrations nor differed between patients with and without DCI. Together with the observed decline in serum creatinine between admission and day 5, these findings suggest that impaired renal clearance is unlikely to be the primary explanation for persistently elevated serum S100B concentrations and instead support ongoing astroglial injury accompanying secondary brain injury.

Because circulating S100B is rapidly cleared from the peripheral circulation, persistently elevated concentrations are unlikely to reflect only the initial hemorrhagic insult and may instead indicate continued astroglial injury associated with evolving secondary brain injury [19,23].

### 3.5. Neuron-Specific Enolase and Comparative Biomarker Performance

Compared with serum S100B, NSE demonstrated lower discriminative performance for early prediction of DCI. Admission NSE concentrations measured within the first 24 h after ictus did not differ significantly between patients who subsequently developed DCI and those who did not, and ROC analysis showed only modest discriminatory ability.

In contrast, admission serum S100B showed greater discriminative performance for identifying patients who subsequently developed DCI. However, NSE concentrations became significantly higher in patients with DCI during later stages of hospitalization, suggesting that NSE reflects secondary neuronal injury occurring during ischemic progression rather than the early pathophysiological processes preceding DCI. This interpretation is consistent with previous reports [36,71].

These findings likely reflect the distinct biological origins of the two biomarkers. S100B, which is primarily derived from astrocytes, reflects early astroglial injury, BBB disruption, and accompanying neuroinflammatory responses, whereas NSE is considered a marker of neuronal injury that increases during evolving ischemia [72]. The distinct temporal profiles observed in our study indicate that S100B and NSE provide complementary information regarding different phases of secondary brain injury following aSAH. While serum S100B was associated with early injury severity and subsequent DCI, NSE became more informative during later stages, potentially reflecting evolving neuronal injury rather than the initial pathological processes.

Although the present study was not designed to develop or validate a multimarker diagnostic algorithm, the complementary temporal profiles of S100B and NSE suggest that combined longitudinal assessment may improve characterization of secondary brain injury after aSAH. Whether this approach provides clinically meaningful incremental value beyond established clinical, radiological, and physiological monitoring remains unknown and should be evaluated in prospective multicenter studies specifically designed to assess multimarker strategies for DCI detection and monitoring.

### 3.6. Systemic Complications

Patients with DCI and those with unfavorable neurological outcomes experienced systemic complications more frequently, particularly infections and cardiovascular events. These findings are consistent with previous studies demonstrating that extracerebral complications substantially contribute to poor outcomes after aSAH [55,59]. Furthermore, Kashiwazaki et al. and Zhong et al. emphasized that pathological processes initiated during EBI contribute not only to DCI development but also to subsequent infectious complications, highlighting the close interaction between cerebral and systemic injury after aSAH [56,57].

The systemic consequences of aSAH extend beyond the central nervous system and involve complex neuroimmune and neurovascular interactions [73,74,75]. EBI triggers sympathetic activation, systemic inflammatory responses, endothelial dysfunction, and BBB disruption, promoting pathological brain–organ interactions involving the cardiovascular, pulmonary, and peripheral vascular systems. These processes have been associated with pulmonary dysfunction, myocardial injury, arrhythmias, stress-induced cardiomyopathy, and other systemic complications following aSAH [73,74,75].

Supporting this concept, Bergström et al. demonstrated acute systemic endothelial dysfunction after aSAH and reported higher S100B concentrations in non-survivors and patients with neurological deficits [76]. Experimental studies further suggest that S100B-RAGE signaling has been implicated in endothelial glycocalyx degradation, increased vascular permeability, and endothelial dysfunction [73]. However, because these mechanisms were not directly investigated in the present study, they should be considered biologically plausible rather than causal explanations for the observed clinical associations.

Consistent with these observations, serum S100B in our cohort correlated with inflammatory biomarkers and was higher in patients who later developed infectious complications, consistent with greater initial brain injury and a more complicated clinical course, supporting the concept that persistently elevated S100B reflects the overall burden of disease rather than isolated cerebral injury. Although the incidence of infectious complications after aSAH has declined over recent decades, acute kidney injury and thromboembolic complications have become increasingly prevalent [77]. Continued efforts to prevent, recognize, and promptly treat systemic complications therefore remain essential for improving long-term outcomes after aSAH.

### 3.7. Strengths and Limitations

The present study has several strengths including prospective serial longitudinal biomarker sampling, simultaneous serum and CSF S100B measurements, detailed phenotyping of DCI, exploratory assessment of cerebral autoregulation, and time-to-event based analyses accounting for the exact timing of DCI onset. Together, these methodological features enabled comprehensive evaluation of longitudinal biomarker dynamics throughout hospitalization rather than relying solely on isolated admission measurements.

Several limitations should be acknowledged. The single-center design and small sample size limit statistical power and generalizability. The relatively small sample size limited the complexity of multivariable analyses and increased the risk of model overfitting. Accordingly, multivariable models should be regarded as exploratory and hypothesis-generating rather than definitive multivariable predictive models. Larger prospective studies are required to validate these findings.

The study population was highly selected, including only patients who underwent clinically indicated CSF drainage and early aneurysm treatment while excluding patients with irreversible primary brain injury. Consequently, the findings may not be generalizable to the entire spectrum of aSAH, particularly patients with less severe disease who do not require CSF drainage or those with catastrophic EBI.

CSF samples were obtained from either ventricular or lumbar drains, depending on the clinically indicated and available drainage route. Although exploratory analyses did not demonstrate a significant influence of sampling route on CSF S100B concentrations, the limited sample size and unequal distribution of drainage routes substantially reduced statistical power. Because S100B concentrations may vary along the craniospinal axis, particularly in the presence of impaired CSF circulation after aSAH, heterogeneity in sampling location may have contributed to the variability in CSF measurements and reduced the reliability of direct serum–CSF comparisons. The limited sample size and unequal distribution of drainage routes precluded an adequately powered analysis stratified by sampling site. Accordingly, the CSF findings and direct serum–CSF comparisons should be interpreted as exploratory.

Because this was an observational biomarker study without direct assessment of S100B–RAGE signaling, endothelial function, or BBB integrity, the present findings cannot determine whether S100B actively contributes to DCI pathogenesis or primarily represents a bystander marker of injury severity.

Incomplete longitudinal sampling of selected biomarkers and cerebral autoregulation measures reduced the available dataset for some analyses. The limited number of deaths constrained survival analyses and multivariable Cox regression, whereas the absence of an external validation cohort limits confirmation of the observed associations and longitudinal biomarker patterns. Finally, the observational design precludes causal inference regarding the relationships between S100B dynamics, systemic inflammation, and DCI. Consequently, the present findings should be regarded as exploratory and hypothesis-generating. Confirmation in larger prospective multicenter studies incorporating standardized event-based biomarker sampling and external validation will be required before serial S100B monitoring can be considered for routine clinical application.

## 4. Materials and Methods

### 4.1. Patient Selection

The prospective, single-center study included patients treated for aSAH who were admitted to the University Clinical Hospital in Wroclaw between March 2023 and December 2024.

Inclusion criteria were: (1) age 18–70 years; (2) admission within 24 h of symptom onset; (3) patients with CSF drainage; and (4) surgical or endovascular aneurysm occlusion within 24 h of admission.

Exclusion criteria were: (1) non-aneurysmal subarachnoid hemorrhage; (2) pre-existing central nervous system disease; (3) patients with acute or chronic infections at admission; (4) intraoperative complications; (5) evidence of irreversible brain injury on admission (including bilaterally fixed and dilated pupils); (6) pregnancy; (7) pre-existing conditions that could affect S100B protein levels (e.g., active malignancy, advanced chronic kidney disease); (8) patient refusal to participate in the study; (9) incomplete clinical, radiological, or laboratory data; and (10) admission more than 24 h after bleeding.

Patients were diagnosed using cranial computed tomography (CT), computed tomography angiography (CTA), or digital subtraction angiography (DSA) and were managed according to a standardized institutional neurocritical care protocol aligned with the European Stroke Organisation (ESO) recommendations and successive updates of the American Heart Association/American Stroke Association (AHA/ASA) guidelines for aSAH [78,79] (see Appendix A.1 for additional details). The cornerstone of management was early aneurysm securing, performed as soon as permitted by the patient’s clinical condition and aneurysm morphology using either microsurgical clipping or endovascular coiling. In selected patients, lumbar CSF drainage was used to facilitate the clearance of blood degradation products from the subarachnoid space and reduce the risk of DCI. In patients with communicating hydrocephalus, intermittent lumbar CSF drainage was additionally performed to reduce intracranial pressure. Patients without hydrocephalus underwent continuous lumbar drainage based on the principles of the EARLYDRAIN protocol, whereas external ventricular drainage (EVD) was reserved primarily for patients with acute severe hydrocephalus [80,81,82,83,84,85,86].

Routine management in the neurocritical care unit included nimodipine administration, maintenance of euvolemia, daily neurological assessments, serial cranial CT examinations, and daily transcranial Doppler (TCD) ultrasonography. Advanced neuromonitoring, including intracranial pressure and cerebral perfusion pressure monitoring as well as cerebral oximetry using near-infrared spectroscopy, together with respiratory and hemodynamic support, was implemented according to individual clinical requirements. Additional pharmacological therapies were administered according to institutional practice. Cerebrolysin was used in selected patients as part of local clinical management but was not included in outcome analyses. Although Cerebrolysin is not specifically recommended in guidelines for aSAH, its use has been supported in European neurorehabilitation recommendations as an adjunctive therapy in early recovery after moderate-to-severe acute ischemic stroke [87].

In patients with elevated cerebral blood flow velocities suggestive of vasospasm, CTA was performed to confirm the diagnosis and guide further management. Treatment of DCI included hemodynamic augmentation with induced hypertension while maintaining euvolemia and, when clinically indicated, endovascular rescue therapy with balloon angioplasty and intra-arterial nimodipine administration. Routine cardiac evaluation during the acute phase of aSAH included electrocardiography and transthoracic echocardiography to detect neurocardiogenic complications.

### 4.2. Definitions

TCD-defined vasospasm was diagnosed according to established criteria, including a mean cerebral blood flow velocity (CBFV) in the middle cerebral artery exceeding 120 cm/s or an approximately 50% daily increase in CBFV [88].

Angiographic vasospasm was defined as the occurrence of new arterial vessel narrowing on follow-up four-vessel DSA or CTA [89].

DCI was defined according to the consensus criteria proposed by Vergouwen et al. as the occurrence of new focal neurological deficits or a decrease of ≥2 points in the Glasgow Coma Scale lasting for at least 1 h, and/or new cerebral infarction on follow-up CT or MRI not attributable to other causes [90].

Dynamic cerebral autoregulation (dCA) was assessed using two complementary indices: the Mxa and the ARI. Both parameters were calculated using the ICM+ software platform, version 9.2.1 (Cambridge Enterprise Ltd., Cambridge, UK) from simultaneous recordings of middle cerebral artery mean blood flow velocity obtained by TCD ultrasonography and arterial blood pressure.

The modified Mxa represents the correlation coefficient between mean cerebral blood flow velocity and arterial blood pressure. Values range from −1 to 1, with higher positive values indicating impaired autoregulatory function. Impaired autoregulation was defined according to previously published thresholds.

The ARI, ranging from 0 to 9, was derived from transfer function analysis of spontaneous fluctuations in arterial blood pressure and cerebral blood flow velocity. Values between 4 and 7 were considered to represent preserved autoregulatory function [91,92,93,94]. Arterial partial pressure of carbon dioxide, an important determinant of cerebrovascular tone, was continuously monitored and maintained within the physiological range throughout the study (see Appendix A.2). Dynamic cerebral autoregulation assessment was performed in accordance with the recommendations of the International Cerebral Autoregulation Research Network white paper [95].

Cardiac complications were defined by the presence of new electrocardiographic changes, elevated cardiac troponin levels, and/or newly detected abnormalities on echocardiography [96]. Infection was defined as any newly diagnosed infectious condition occurring after admission to the intensive care unit (ICU) [97].

### 4.3. Data Collection

Baseline demographic characteristics, premorbid medical history, and clinical data were collected for all patients. Neurological status on admission was assessed using the GCS and the World Federation of Neurosurgical Societies (WFNS) grading system. Patients with WFNS grades I–III were classified as having good-grade aSAH, whereas those with grades IV–V were classified as having poor-grade disease [98].

Radiographic severity was evaluated using the modified Fisher scale [99], and early brain edema was assessed with the SEBES [100]. DCI risk stratification was additionally performed using the VASOGRADE classification [101,102]. Systemic disease severity at ICU admission was assessed using the Acute Physiology and Chronic Health Evaluation II (APACHE II) score.

Additional variables included aneurysm location, presence of intraparenchymal hematoma, aneurysm treatment modality, requirement for CSF diversion, strategy and systemic complications occurring during the ICU stay.

Functional outcome was assessed at hospital discharge and 3 months after hemorrhage onset using the modified Rankin Scale (mRS). Favorable outcome was defined as a mRS score of 0–2, whereas scores of 3–6 were considered unfavorable [103]. Three-month outcome assessment was performed by an experienced intensive care physician using a standardized questionnaire during an outpatient visit or a structured telephone interview.

### 4.4. Sample Collection

All biomarker samples were prospectively collected according to a predefined sampling schedule and analyzed using standardized laboratory procedures. CRP, WBC, PCT and lactate were measured daily. IL-6 was measured according to the study protocol at predefined sampling time points. The NLR was calculated as the absolute neutrophil count divided by the absolute lymphocyte count, whereas the SII was calculated as platelet count × neutrophil count / lymphocyte count according to the method described by Bo Hu et al. [63].

Serum NSE concentrations were measured on days 1 and 5 using an automated chemiluminescent immunoassay system (Liaison XL; DiaSorin, Saluggia, Italy) according to the manufacturer’s instructions. Serum and CSF S100B concentrations were measured within 24 h after admission and subsequently on days 3, 5, 7, and 10. CSF samples were obtained according to clinical indications by lumbar puncture or from lumbar or ventricular drainage systems when available. The route of CSF sampling was determined by routine clinical management rather than by the study protocol, whereas blood samples were collected from central venous catheters. Samples were immediately processed and stored at −80 °C until analysis.

S100B concentrations were measured using a commercially available ELISA kit (Human S100B DuoSet, DY1820-05; R&D Systems, Minneapolis, MN, USA) according to the manufacturer’s instructions. The assay had a working range of 46.9–3000 pg/mL. Biomarker analyses were performed either in the Department of Chemistry and Immunochemistry, Medical University of Wroclaw, Poland, or as part of routine clinical diagnostics.

Biomarker analyses were performed after completion of clinical follow-up and outcome adjudication.

### 4.5. Statistical Analysis

Statistical analyses were performed using R statistical software version 4.5.2 (R Foundation for Statistical Computing, Vienna, Austria). A two-sided significance level of α = 0.05 was applied throughout all analyses. Due to the relatively small sample size and predominantly non-normal distribution of variables, continuous data are presented as median and interquartile range (IQR), whereas categorical variables are reported as counts and percentages.

Baseline comparisons between patients with and without DCI, as well as between favorable and unfavorable 3-month functional outcomes, were performed using the Wilcoxon rank-sum test for continuous variables and Fisher’s exact test for categorical variables. Effect sizes for continuous variables were estimated using Wilcoxon r and interpreted according to conventional thresholds (0.1 small, 0.3 moderate, 0.5 large). For categorical analyses, Cramér’s V was calculated where appropriate. For patients who developed DCI, each serum S100B measurement was classified as occurring before (Pre-DCI), on (Peri-DCI), or after (Post-DCI) the day of DCI onset. Linear mixed-effects models with patient-specific random intercepts were fitted to compare longitudinal serum S100B concentrations between DCI phases and between patients with and without DCI while accounting for repeated measurements and hospital day. An interaction between DCI status and hospital day was additionally evaluated to assess whether longitudinal trajectories differed between groups.

*p*-values from the correlation analyses between admission serum S100B and inflammatory biomarkers were additionally adjusted for multiple comparisons using the Benjamini–Hochberg false discovery rate (FDR) procedure.

Receiver operating characteristic (ROC) curve analyses were performed to evaluate the predictive performance of serum S100B concentrations for DCI occurrence and unfavorable 3-month neurological outcome. Area under the curve (AUC), optimal cutoff values based on the Youden index, sensitivity, specificity, and overall classification performance were calculated. Separate ROC analyses were additionally performed for peak longitudinal S100B concentrations during hospitalization. Longitudinal biomarker dynamics were analyzed using linear mixed-effects models with random intercepts only for individual patients to account for repeated measurements obtained during hospitalization. Fixed effects included hospitalization day, DCI occurrence, TCD-defined vasospasm, and functional outcome group, together with interaction terms where appropriate. Biomarkers analyzed longitudinally included serum S100B, CRP, and WBC. Serum S100B concentrations were log-transformed prior to modeling to improve distributional properties and model stability. Longitudinal trajectories were additionally visualized using repeated-measures trajectory plots. Time-to-event analyses were performed to investigate the association between admission serum S100B concentrations and time to DCI occurrence. Kaplan–Meier survival curves were generated using ROC-derived S100B thresholds, and differences between groups were assessed using the log-rank test. Cox proportional hazards regression models were used to estimate hazard ratios (HRs) with corresponding 95% confidence intervals (CIs).

To assess potential confounding effects, additional linear mixed-effects models were fitted including infectious complications and treatment modality (microsurgical clipping vs. endovascular coiling) as fixed effects together with sampling day, while accounting for repeated measurements within patients.

Correlation analyses were additionally performed to investigate associations between serum S100B concentrations and markers of disease severity, neuroinflammation, vasospasm burden, autoregulatory dysfunction, and renal function. Spearman rank correlation coefficients were calculated for relationships between S100B and clinical severity scales (APACHE II, GCS, motor GCS, Hunt–Hess grade, WFNS, Fisher scale, SEBES scale, and VASOGRADE classification), inflammatory biomarkers (CRP, IL-6, PCT, NLR, SII, and WBC), cerebral autoregulation indices (Mxa and ARI), and serum creatinine concentrations.

To explore the potential influence of renal clearance on serum S100B kinetics, additional analyses evaluated the relationship between admission serum creatinine concentration, longitudinal creatinine changes, and serum S100B levels. Admission serum creatinine and day-5 serum creatinine were analyzed in relation to admission and longitudinal serum S100B concentrations using correlation analyses and multivariable regression models.

Exploratory analyses evaluated cumulative S100B exposure, peak biomarker concentrations, serum-versus-CSF S100B kinetics, vasospasm timing, and NSE performance. Given the exploratory design and limited sample size, no formal correction for multiple comparisons was applied; therefore, secondary analyses should be interpreted cautiously. Statistical analyses and reporting were conducted in accordance with the STROBE (Strengthening the Reporting of Observational Studies in Epidemiology) recommendations for observational studies and the standards for reporting diagnostic accuracy studies (STARD) 2015 recommendations for analyses of biomarker diagnostic accuracy.

## 5. Conclusions and Future Directions

The present study suggests that longitudinal serum S100B monitoring may provide insight into the temporal evolution of secondary brain injury following aneurysmal subarachnoid hemorrhage. Persistently elevated serum S100B concentrations were associated with delayed cerebral ischemia, vasospasm, systemic inflammatory activation, and unfavorable neurological outcome, whereas admission S100B correlated with established measures of initial hemorrhagic injury.

The similar temporal profiles observed in serum and cerebrospinal fluid support the potential use of peripheral blood sampling as a practical surrogate for intracranial biomarker dynamics, offering a minimally invasive approach to longitudinal neuromonitoring. Together, these findings suggest that serial serum S100B assessment may complement established clinical and radiological risk stratification by providing additional information regarding the temporal evolution of secondary brain injury, rather than replacing existing prognostic tools. Furthermore, S100B should currently be regarded as a complementary biomarker rather than an independent prognostic marker.

Further prospective multicenter studies with larger patient cohorts, standardized longitudinal sampling protocols, and external validation are needed to determine the clinical utility of serial S100B monitoring for identifying patients at increased risk of DCI and unfavorable neurological outcome. Future studies should also establish whether longitudinal S100B assessment provides clinically meaningful incremental value beyond existing multimodal prediction models.

## Figures and Tables

**Table 1:** Baseline demographic, clinical, radiological, and laboratory characteristics of patients with aneurysmal subarachnoid hemorrhage according to delayed cerebral ischemia (DCI) status. Continuous variables are presented as median [interquartile range], and categorical variables as *n* (%). Group comparisons were performed using the Wilcoxon rank-sum test for continuous variables and Fisher’s exact test for categorical variables. GCS, Glasgow Coma Scale; WFNS, World Federation of Neurosurgical Societies; SEBES, Subarachnoid Hemorrhage Early Brain Edema Score; ICU, intensive care unit, hs, high sensitivity; CRP, C-reactive protein; WBC, white blood cell; PLT, platelets; HB, hemoglobin; HT, hematocrit.

Characteristic	Overall *n* = 26 ^1^	No DCI *n* = 10 ^1^	DCI *n* = 16 ^1^	*p*-Value ^2^
Demographic characteristics				
Female sex	15 (58%)	5 (50%)	10 (63%)	0.7
Age, years	54.0 [41.0–61.0]	48.0 [35.0–63.0]	54.5 [46.0–60.5]	0.5
Body mass index	24.7 [23.0–27.7]	25.2 [24.2–29.4]	23.9 [22.0–26.2]	0.12
Hypertension	12 (46%)	4 (40%)	8 (50%)	0.7
Current smoking	17 (65%)	6 (60%)	11 (69%)	0.7
Clinical presentation at admission				
Systolic blood pressure, mmHg	152.0 [130.0–180.0]	142.5 [130.0–158.0]	160.0 [130.0–195.0]	0.3
Mean arterial pressure, mmHg	110.7 [90.0–133.3]	107.0 [90.0–113.7]	116.7 [91.7–140.0]	0.3
Heart rate, bpm	90.0 [62.0–120.0]	79.0 [56.0–120.0]	92.5 [69.5–120.0]	0.6
Loss of consciousness at ictus	19 (73%)	8 (80%)	11 (69%)	0.7
Convulsions at ictus	9 (35%)	1 (10%)	8 (50%)	0.087
Bilaterally reactive pupils	23 (88%)	9 (90%)	14 (88%)	>0.9
Unilaterally reactive pupils	3 (12%)	1 (10%)	2 (13%)	>0.9
Motor GCS < 6	15 (58%)	3 (30%)	12 (75%)	0.043
Glasgow Coma Scale < 13	11 (42%)	3 (30%)	8 (50%)	0.4
Hemorrhage severity and radiological characteristics				
Multiple aneurysms	6 (23%)	2 (20%)	4 (25%)	>0.9
Anterior circulation aneurysm	23 (88%)	8 (80%)	15 (94%)	0.5
APACHE II score	18.5 [12.0–30.0]	11.5 [9.0–20.0]	22.5 [17.0–30.5]	0.045
VASOGRADE red	11 (42%)	3 (30%)	8 (50%)	0.4
SEBES 3–4	9 (35%)	2 (20%)	7 (44%)	0.4
Hunt-Hess 4–5	10 (38%)	3 (30%)	7 (44%)	0.7
Modified Fisher 4	15 (58%)	4 (40%)	11 (69%)	0.2
WFNS 4–5	11 (42%)	3 (30%)	8 (50%)	0.4
Intracerebral hemorrhage	7 (27%)	3 (30%)	4 (25%)	>0.9
Laboratory parameters				
Hb [g/dL] (14–18)	13.2 [12.4–14.9]	13.5 [12.4–14.2]	13.1 [12.2–15.0]	>0.9
WBC [G/L] (4–10)	14.9 [11.0–17.1]	13.3 [10.5–17.1]	15.1 [11.8–17.2]	0.4
Ht [%] (40–54)	39.3 [36.6–42.4]	40.8 [36.9–43.2]	38.3 [35.4–42.3]	0.8
PLT [10^3^/uL] (140–440)	250.5 [225.0–307.0]	255.0 [233.0–307.0]	250.5 [222.5–302.5]	>0.9
Troponin, pg/mL (0–34.2)	31.4 [2.3–119.8]	5.9 [1.9–35.5]	53.7 [11.7–251.2]	0.067
CRP, [mg/L] (0–5)	2.6 [1.3–6.8]	3.5 [0.7–5.5]	2.4 [1.6–7.0]	>0.9
Bilirubin, mg/dL (0.2–1.2)	0.6 [0.4–0.8]	0.7 [0.5–0.8]	0.6 [0.4–0.7]	0.3
Creatinine, mg/dL (0.8–1.3)	0.7 [0.7–0.9]	0.7 [0.7–0.8]	0.7 [0.6–0.9]	0.9
Treatment characteristics				
Surgical clipping	4 (15%)	4 (40%)	0 (0%)	0.014
Endovascular treatment	22 (85%)	6 (60%)	16 (100%)	0.014
Time from admission to neurosurgical intervention, h	3.5 [2.3–4.6]	3.5 [2.5–9.3]	3.4 [2.1–4.0]	0.4

^1^*n* (%); Median [Q1–Q3]. ^2^ Fisher’s exact test; Wilcoxon rank sum test; Wilcoxon rank sum exact test.

**Table 2:** Clinical outcomes, vasospasm characteristics, autoregulatory disturbances, and in-hospital complications according to delayed cerebral ischemia (DCI) status. Continuous variables are presented as median [interquartile range], and categorical variables as *n* (%). Group comparisons were performed using the Wilcoxon rank-sum test for continuous variables and Fisher’s exact test for categorical variables. Horowitz index represents the PaO2/FiO2 ratio. LOS = length of stay; ICU = intensive care unit; TCD = transcranial Doppler; CSF = cerebrospinal fluid; DCI = delayed cerebral ischemia; mRS = modified Rankin Scale.

Characteristic	Overall *n* = 26 ^1^	No DCI *n* = 10 ^1^	DCI *n* = 16 ^1^	*p*-Value ^2^
Clinical Outcomes				
Poor discharge outcome (mRS 3–6)	19 (73%)	4 (40%)	15 (94%)	0.005
Poor 3-month outcome (mRS 3–6)	16 (62%)	3 (30%)	13 (81%)	0.015
Focal neurological deficit	13 (65%)	4 (40%)	9 (90%)	0.057
TCD vasospasm	15 (58%)	0 (0%)	15 (94%)	<0.001
CT/angiographic vasospasm	15 (58%)	2 (20%)	13 (81%)	0.004
Delayed cerebral ischemia (DCI)	16 (62%)	0 (0%)	16 (100%)	<0.001
Time to DCI, days	5.0 [3.0–8.0]	NA [NA–NA]	5.0 [3.0–8.0]	
Hospital Course				
ICU length of stay, days	13.0 [9.0–19.0]	10.0 [9.0–16.0]	14.5 [10.5–19.0]	0.2
Hospital length of stay, days	20.0 [14.0–37.0]	20.0 [17.0–37.0]	19.5 [14.0–35.0]	0.6
Mechanical ventilation duration, days	6.0 [2.0–14.0]	1.5 [0.0–9.0]	8.0 [3.5–14.0]	0.089
Systemic complications				
Horowitz index (PaO2/FiO2)	292.0 [217.0–406.0]	353.5 [181.0–463.0]	280.5 [222.0–377.5]	0.5
Pulmonary edema	5 (19%)	3 (30%)	2 (13%)	0.3
Cardiovascular complications	17 (65%)	5 (50%)	12 (75%)	0.2
Any infection	13 (50%)	2 (20%)	11 (69%)	0.041
Multiple organ failure	2 (7.7%)	0 (0%)	2 (13%)	0.5
Infectious complications				
Hospital-acquired pneumonia	10 (38%)	2 (20%)	8 (50%)	0.2
Urinary tract infection	7 (27%)	1 (10%)	6 (38%)	0.2
Bloodstream infection	1 (3.8%)	0 (0%)	1 (6.3%)	>0.9
CSF infection	1 (3.8%)	0 (0%)	1 (6.3%)	>0.9

^1^*n* (%); Median [Q1–Q3]. ^2^ Fisher’s exact test; NA; Wilcoxon rank sum test; Wilcoxon rank sum exact test.

## Data Availability

Anonymized data may be made available from the corresponding author upon reasonable request.

## Abbreviations

The following abbreviations are used in this manuscript:

S100B	S100 calcium-binding protein B
aSAH	aneurysmal subarachnoid hemorrhage
CSF	cerebrospinal fluid
DCI	delayed cerebral ischemia
AUC	area under the curve
EBI	early brain injury
BBB	blood–brain barrier
RAGE	receptor for advanced glycation end products
DAMP	damage-associated molecular pattern
IL-6	interleukin-6
APACHE II	Acute Physiology and Chronic Health Evaluation II
GCS	Glasgow Coma Scale
IQR	interquartile range
mRS	modified Rankin Scale
WFNS	World Federation of Neurosurgical Societies
SEBES	Subarachnoid Hemorrhage Early Brain Edema Score
ICU	intensive care unit
WBC	white blood cell count
PLT	platelets
Hb	hemoglobin
Ht	hematocrit
TCD	transcranial Doppler
LOS	length of stay
HR	hazard ratio
NSE	neuron-specific enolase
ROC	receiver operating characteristic
CI	confidence interval
NLR	neutrophil-to-lymphocyte ratio
CRP	C-reactive protein
PCT	procalcitonin
SII	systemic immune-inflammation index
ARI	autoregulation index
Mxa	modified mean flow index
TOx	tissue oxygenation index
CT	computed tomography
CTA	computed tomography angiography
DSA	digital subtraction angiography
ESO	European Stroke Organisation
AHA/ASA	American Heart Association/American Stroke Association
EVD	external ventricular drainage
CBFV	cerebral blood flow velocity
CV	cerebral vasospasm
DIND	delayed ischemic neurologic deficit
MR	magnetic resonance
dCA	dynamic cerebral autoregulation
ELISA	enzyme-linked immunosorbent assay

## Appendix A

### Appendix A.1. Neurocritical Care Management

All patients were treated according to a standardized institutional neurocritical care protocol aligned with the European Stroke Organisation (ESO) recommendations and the American Heart Association/American Stroke Association (AHA/ASA) guidelines for aneurysmal subarachnoid hemorrhage (aSAH) [78,79]. Early aneurysm occlusion was performed using either microsurgical clipping or endovascular coiling.

Cerebrospinal fluid (CSF) diversion was applied according to clinical indications. Continuous lumbar drainage was used in patients without hydrocephalus according to the EARLYDRAIN protocol, whereas intermittent lumbar drainage was performed in communicating hydrocephalus. External ventricular drainage was reserved for patients with acute severe hydrocephalus [80,81,82,83,84,85,86].

Routine neurocritical care included nimodipine administration, multimodal neuromonitoring, serial neuroimaging, daily transcranial Doppler (TCD) examinations, and supportive therapy according to institutional protocols. Additional neuroprotective therapy, including Cerebrolysin, was administered according to institutional practice [88].

Patients with suspected vasospasm underwent computed tomography angiography (CTA). DCI was managed using induced hypertension while maintaining euvolemia and, when indicated, endovascular rescue therapy with balloon angioplasty and intra-arterial nimodipine.

### Appendix A.2. Definitions and Outcome Measures

#### Appendix A.2.1. Delayed Cerebral Ischemia

DCI was defined according to the multidisciplinary consensus criteria proposed by Vergouwen et al. [90] as either:

• delayed ischemic neurological deficit (DIND), defined as new focal neurological impairment or a decrease of at least two points on the Glasgow Coma Scale (GCS) lasting ≥1 h and not explained by other causes; and/or
• cerebral infarction detected on follow-up computed tomography (CT) or magnetic resonance imaging (MRI) within 6 weeks after aSAH that was absent on imaging performed 24–48 h after aneurysm occlusion and could not be attributed to surgical or endovascular treatment, ventricular catheter placement, intraparenchymal hematoma, or other non-ischemic causes.

#### Appendix A.2.2. Vasospasm

TCD-defined vasospasm was defined as a mean middle cerebral artery blood flow velocity > 120 cm/s or an approximately 50% increase compared with the previous examination [88]. Angiographic vasospasm was defined as new arterial narrowing detected on follow-up digital subtraction angiography (DSA) or computed tomography angiography (CTA) [89].

#### Appendix A.2.3. Dynamic Cerebral Autoregulation

Dynamic cerebral autoregulation (dCA) was assessed using the modified mean flow index (Mxa) and autoregulation index (ARI), calculated with ICM+ software (Cambridge Enterprise Ltd., Cambridge, UK) from simultaneous recordings of arterial blood pressure and middle cerebral artery blood flow velocity. Impaired autoregulation was defined as Mxa > 0.30, whereas ARI values of 4–7 indicated preserved autoregulatory function [92,93,94,95]. Arterial carbon dioxide tension (PaCO_2_) was maintained within the physiological range during monitoring. Autoregulation assessment followed the recommendations of the International Cerebral Autoregulation Research Network (CARNet) [96].

#### Appendix A.2.4. Systemic Complications and Functional Outcome

Cardiac complications were defined according to previously published criteria [96]. Infection was defined as any newly diagnosed infectious condition after intensive care unit (ICU) admission [97]. Functional outcome was assessed using the modified Rankin Scale (mRS); scores of 0–2 were considered favorable and scores of 3–6 unfavorable [103].
